# *Halomonas ventosae* JPT10 promotes salt tolerance in foxtail millet (*Setaria italica*) by affecting the levels of multiple antioxidants and phytohormones

**DOI:** 10.1002/pei3.10122

**Published:** 2023-09-11

**Authors:** Shenghui Xiao, Yiman Wan, Yue Zheng, Yongdong Wang, Jiayin Fan, Qian Xu, Zheng Gao, Changai Wu

**Affiliations:** ^1^ National Key Laboratory of Wheat Improvement, Shandong Engineering Research Center of Plant‐Microbial Restoration for Saline‐Alkali Land, College of Life Sciences Shandong Agricultural University Tai'an Shandong province China

**Keywords:** foxtail millet, *Halomonas ventosae*, JA, metabolome, OPDA, phytohormone, salt tolerance

## Abstract

Plant growth‐promoting bacterias (PGPBs) can increase crop output under normal and abiotic conditions. However, the mechanisms underlying the plant salt tolerance‐promoting role of PGPBs still remain largely unknown. In this study, we demonstrated that *Halomonas ventosae* JPT10 promoted the salt tolerance of both dicots and monocots. Physiological analysis revealed that JPT10 reduced reactive oxygen species accumulation by improving the antioxidant capability of foxtail millet seedlings. The metabolomic analysis of JPT10‐inoculated foxtail millet seedlings led to the identification of 438 diversely accumulated metabolites, including flavonoids, phenolic acids, lignans, coumarins, sugar, alkaloids, organic acids, and lipids, under salt stress. Exogenous apigenin and chlorogenic acid increased the salt tolerance of foxtail millet seedlings. Simultaneously, JPT10 led to greater amounts of abscisic acid (ABA), indole‐3‐acetic acid (IAA), salicylic acid (SA), and their derivatives but lower levels of 12‐oxo‐phytodienoic acid (OPDA), jasmonate (JA), and JA‐isoleucine (JA‐Ile) under salt stress. Exogenous JA, methyl‐JA, and OPDA intensified, whereas ibuprofen or phenitone, two inhibitors of JA and OPDA biosynthesis, partially reversed, the growth inhibition of foxtail millet seedlings caused by salt stress. Our results shed light on the response of foxtail millet seedlings to *H. ventosae* under salt stress and provide potential compounds to increase salt tolerance in foxtail millet and other crops.

## Graphical abstract

We demonstrated that *Halomonas ventosae* JPT10 promoted the salt tolerance of both dicots and monocots. JPT10 could improve the antioxidant capability of foxtail millet and the levels of abscisic acid, indole‐3‐acetic acid, salicylic acid, and their derivatives but reduce the levels of 12‐oxo‐phytodienoic acid (OPDA), jasmonate (JA), and JA‐isoleucine (JA‐Ile).

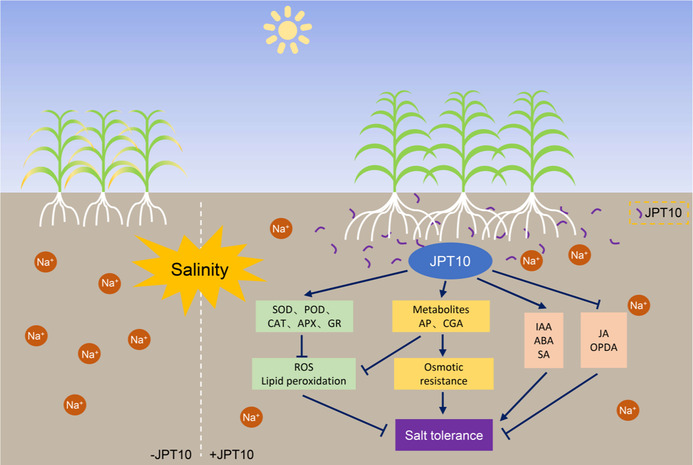

## 1 INTRODUCTION

Plant growth‐promoting bacterias (PGPBs) increase the production of different crops and their abilities to withstand biotic and abiotic stressors. Researchers have been drawn to creating biotechnological methods that use PGPBs to enhance the growth of stressed plants. PGPBs from naturally salty environments could provide real candidates that more efficiently lessen the impacts of salt stress (Masmoudi et al., 2019). For example, halotolerant *Bacillus licheniformis* HSW‐16 causes wheat (*Triticum aestivum*) to develop systemic salt tolerance (Singh & Jha, 2016). *Porostereum spadiceum* AGH786 can greatly improve wheat growth under salt stress (Gul et al., 2022). Moreover, chickpea growth under elevated salt stress was boosted by injection with *Halomonas variabilis* HT1 and *Planococcus rifietoensis* RT4 (Qurashi & Sabri, 2012). However, very little is known about the mechanism underlying *Halomonas ventosae*‐mediated plant salt tolerance.

PGPBs flourishing in the rhizosphere promote plant growth through a number of processes (Ali et al., 2021, 2022; Bibi et al., 2019; Ikram et al., 2019; Jan et al., 2019; Mishra et al., 2018; Wang et al., 2022). These mechanisms include increased nutrient uptake, induced systemic resistance, and the generation of phytohormones, siderophores, antioxidants, exopolysaccharides (EPS), osmoprotectants, and enzymes, such as 1‐aminocyclopropane‐1‐carboxylate (ACC) deaminase (Numan et al., 2018). By decreasing ethylene synthesis near the growing roots, PGPBs with ACC deaminase activity, for example, have been utilized to successfully mitigate the adverse effects of salinity (Glick et al., 2007; Orozco‐Mosqueda et al., 2020). By producing ACC deaminase and IAA, *Streptomyces venezuelae* ATCC 10712 and *Enterobacter* sp. EJ01 improved the salt tolerance of Thai jasmine rice (*Oryza sativa* L. cv. ‘KDML105’), tomato, and *Arabidopsis* (Kim et al., 2014; Yoolong et al., 2019). The salt tolerance of pear millet was improved by the endophyte *Aspergillus terreus* by improving the production of IAA and secondary metabolites (Khushdil et al., 2019). *P. spadiceum* AGH786 boosted the salt tolerance and growth of soybean seedlings by modulating endogenous phytohormones and isoflavones (Hamayun et al., 2017). *Aspergillus flavus* CSH1 improves soybean growth and salt tolerance by decreasing ABA and JA production (Lubna et al., 2018). *Yarrowia lipolytica* inoculation promotes maize growth through controlling metabolism and hormonal (such as ABA and IAA) secretions under salinity (Gul Jan et al., 2019). *A. terreus* BTK‐1 inoculation have promoted wheat against salinity (Khan et al., 2022). In salt‐stressed maize, *Staphylococcus sciuri* SAT‐17 mediates antioxidative defense and growth modulation (Akram et al., 2016; Yasin et al., 2018). Proline and total soluble sugar levels were higher in *Bacillus amyloliquefaciens‐*inoculated rice seedlings than in non‐inoculated seedlings (Tiwari et al., 2017). Furthermore, chickpea plants inoculated with *Azosprillum lipoferum* showed accumulation of soluble sugars, glycine betaine, proline, and other salt‐stress proteins (El‐Esawi et al., 2019). However, it is unclear whether salt stress‐related antioxidants, osmoprotectants, and hormone changes are mediated by *H. ventosae*.

In this study, we performed a series of physiological experiments to explain whether and how halophilic *H. ventosae* JPT10 promotes plant salt tolerance. We found that JPT10 promoted salt tolerance in both dicots and monocots, namely foxtail millet, maize, wheat, soybean, and tomato. Under salt stress, JPT10 increased the antioxidant capacity by the accumulation of apigenin (AP), chlorogenic acid (CGA), and other antiocidants, increasing the activity of important antioxidant enzymes, as well as ABA, IAA, and SA content but decreasing the levels of JA, OPDA, and JA‐Ile. Furthermore, exogenous AP, CGA, and inhibitors of JA and OPDA biosynthesis partially reversed the growth inhibition caused by salt stress. As a result, our findings provide potential active substances for improving salt tolerance in crops, and offer physiological mechanisms for JPT10‐mediated salt tolerance of foxtail millet and other crops.

## 2 RESULTS

### 2.1 JPT10 promotes salt tolerance of foxtail millet seedlings

**FIGURE 1:**
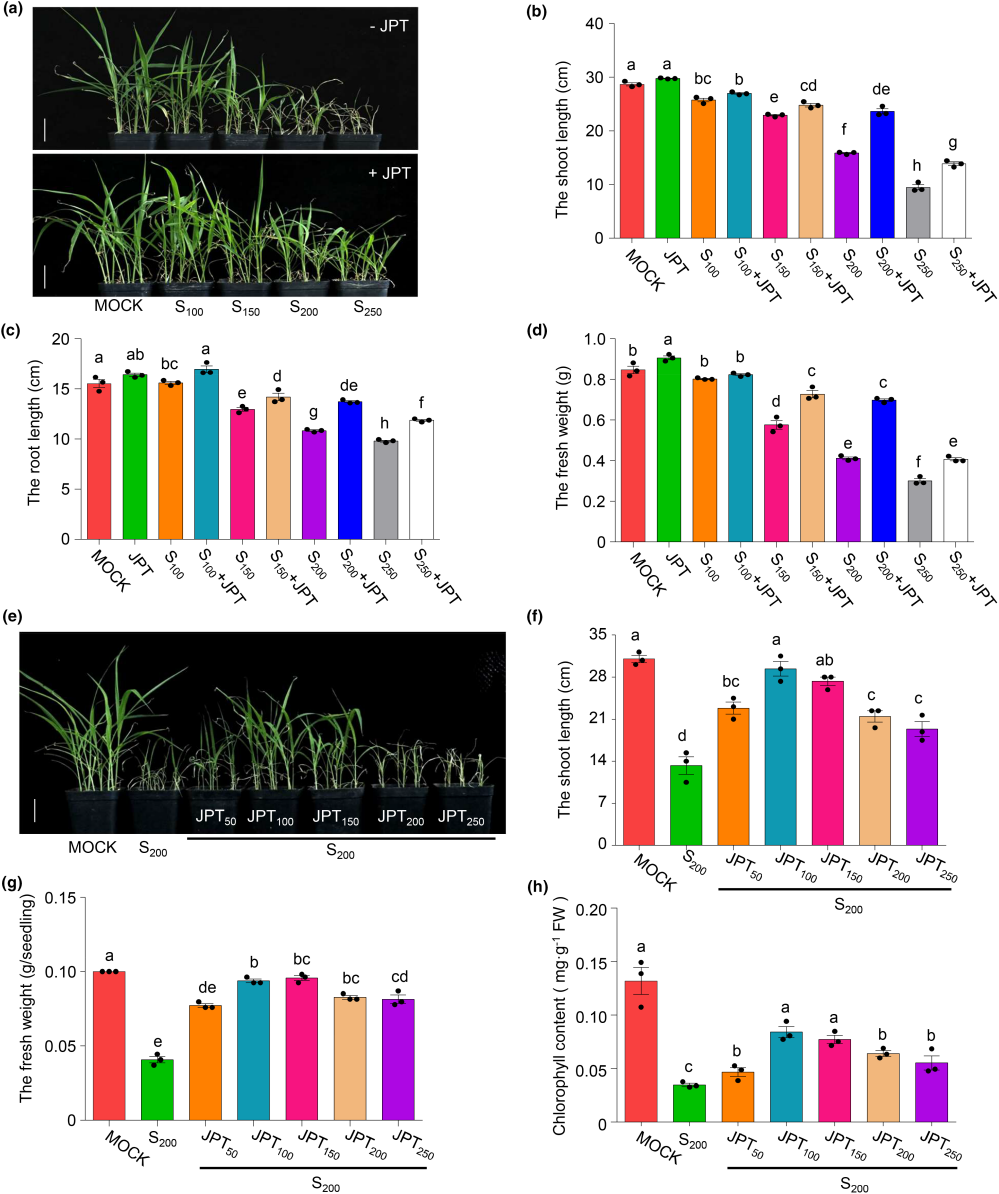
JPT10 promotes salt tolerance of foxtail millet seedlings. (a) 21‐day‐old foxtail millet seedlings treated with different NaCl concentrations in the presence or absence of JPT10, respectively. S_n_ indicated 100, 150, 200, or 250 mM NaCl. Seedlings treated with sterile water were used as control (MOCK). Scale bars = 5 cm. Two liters of the indicated concentrations of NaCl solution was irrigated in the NaCl‐treated groups. For the “+JPT” groups, 200 mL JPT10 solution at the concentration of 10^10^ CFU/mL was added to the 2 L of the indicated concentrations of NaCl solution. The “−JPT” indicated no JPT10 was added. Plants were treated every 3 days and treated 3 times. (b–d) The shoot length, root length and fresh weight of foxtail millet seedlings treated as described in (a). Error bars indicate SEM (*n* = 3). (*p* ≤ .05; one‐way ANOVA). (e) 21‐day‐old foxtail millet seedlings treated with 200 mM NaCl in the presence or absence of the indicated concentrations of JPT10, respectively. The indicated volumes of JPT10 (JPT_50_, JPT_100_, JPT_150_, JPT_200_, JPT_250_) at the concentration of 10^10^ CFU/mL were mixed with 2 L of 200 mM NaCl (S_200_) solution before irrigation. Seedlings treated with sterile water were used as control (MOCK). Scale bars = 5 cm. Plants were treated every 3 days and treated for 3 times. (f–h) The shoot length, fresh weight and chlorophyll content of foxtail millet seedlings treated as described in (e). Error bars indicate SEM (*n* = 3). (*p* ≤ .05; one‐way ANOVA).

To investigate the impact of JPT10 on the performance of foxtail millet seedlings under salt stress, foxtail millet seedlings were watered with various concentrations of NaCl solution with or without JPT10. After 21 days of salt exposure, foxtail millet seedlings showed slow growth and senescence (Figure 1a). Both the fresh weight and the length of the shoots and roots dramatically decreased (Figure 1b–d). By contrast, the shoot length of JPT10‐inoculated foxtail millet seedlings increased by roughly 1.4 and 1.2 times, respectively, in the presence of 200 and 250 mM NaCl (Figure 1b). A similar promotion was obtained in the root length and fresh weight (Figure 1c,d). Furthermore, under salt stress, the early seedling growth was also considerably enhanced by JPT10 (Figure S1). These results demonstrate that JPT10 can effectively alleviates the salt stress of foxtail millet seedlings.

Different JPT10 concentrations were utilized in the presence of 200 mM NaCl to determine the optimal concentration of JPT10. When treated with a mixture of NaCl and various concentrations of JPT10, foxtail millet seedlings grew better than when treated with just NaCl, and the best seedling growth with the longest roots, highest fresh weight, and highest chlorophyll content was obtained when inoculated with 100 mL JPT10 (Figure 1e–h. Interestingly, there were no noticeable changes in growth between JPT10‐inoculated and non‐inoculated seedlings when 200 mM NaCl was replaced by lowering concentrations of NaCl mixed with increasing concentrations of NaHCO_3_ (Figure S2). These results indicate that JPT10 effectively alleviates salt but not alkaline stress in foxtail millet seedlings.

### 2.2 JPT10 increases antioxidant ability in foxtail millet seedlings

**FIGURE 2:**
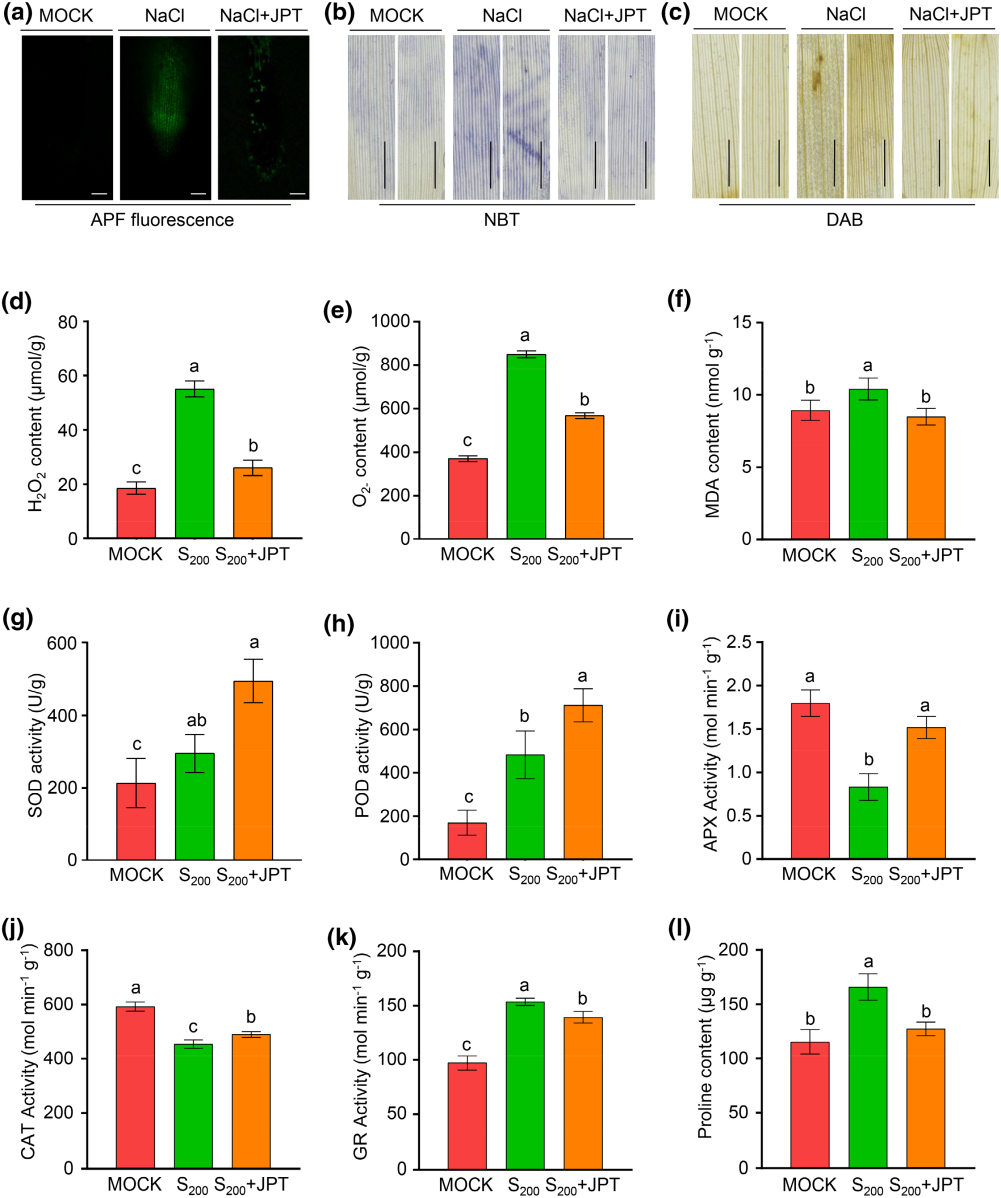
JPT10 increases antioxidant ability of foxtail millet seedlings. (a–c) APF fluorescence in root tips (a) and nitrotetrazolium (NBT) (b) and diaminobenzidine (DAB) (c) staining in leaves to indicate the contents of H_2_O_2_ and O_2_^−^ in foxtail millet seedlings treated with 200 mM NaCl (S_200_) in the presence or absence of JPT10, respectively. Seedlings treated with sterile water were used as control (MOCK). Scale bars = 1 cm. (d–l) Determination of H_2_O_2_ (d), O_2_^−^ (e) and malondialdehyde (MDA) (f) contents, enzyme activities of superoxide dismutase (SOD) (g), peroxidase (POD) (h), ascorbate peroxidase (APX) (i), catalase (CAT) (j), and glutathione reductase (GR) (kK), and proline content (l) in foxtail millet seedlings treated with 200 mM NaCl in the presence or absence of JPT10, respectively. Three biological repeats were performed (*p* ≤ .05; one‐way ANOVA).

The impact of JPT10 on reactive oxygen species (ROS) homeostasis was measured to determine how it boosts the salt tolerance of foxtail millet. The ROS levels were determined by aminophenyl fluorescein (APF), a fluorescence probe for hydroxyl radical and peroxynitrite (Tamura et al., 2023). APF fluorescence signal was higher in salt stressed foxtail millet seedlings than the control, while that was lower in salt stressed foxtail millet seedling in the presence of JPT10 (Figure 2a), indicating a lower ROS accumulation of JPT10‐inoculated foxtail millet leaves than the control. Similarly, JPT10‐inoculated foxtail millet leaves produced low diaminobenzidine (DAB) and nitrotetrazolium blue chloride (NBT) dying under salt stress, salt stress alone caused deep DAB and NBT dying, indicating a lower H_2_O_2_ and O_2_^−^ accumulation of JPT10‐inoculated foxtail millet leaves than the control (Figure 2b,c). Consistently, foxtail millet seedlings had higher H_2_O_2_ and O_2_^−^ contents than JPT10‐inoculated seedlings when exposed to salt stress (Figure 2d,e). The contents of the membrane lipid peroxidation product malondialdehyde (MDA) were higher under salt stress but not changed under the combination of salt stress and JPT10 (Figure 2f). These data indicate that JPT10 reduces ROS buildup in foxtail millet seedlings that are experiencing salt stress. To further evaluate the effect of the JPT10 inoculation on foxtail millet under salt stress, we determined activities of several important antioxidant enzymes. In foxtail millet seedlings, salt stress and a combination of salt stress and JPT10 elevated superoxide dismutase (SOD) and peroxidase (POD) activities. However, foxtail millet seedlings had stronger SOD and POD activities under salt stress in conjunction with JPT10 than with salt stress alone (Figure 2g,h). The activity of APX (ascorbate peroxidase) was significantly reduced in foxtail millet seedlings under salt stress but was increased comparably to the control under the combination of salt stress and JPT10 (Figure 2i). The activity of CAT (catalase) was significantly reduced in foxtail millet seedlings under both salt stress and the combination of salt stress and JPT10 compared to the control (Figure 2j). Whereas, the activity of GR (glutathione reductase) was significantly increased in foxtail millet seedlings under both salt stress and the combination of salt stress and JPT10 compared to the control (Figure 2k). However, the proline content was not increased by JPT10 under salt stress (Figure 2l). These results suggest that JPT10 can help foxtail millet seedlings maintain high antioxidant enzyme activity.

Moreover, we detected the levels of antioxidants using a metabolomic platform that combined ultra‐high‐performance liquid chromatography and tandem mass spectrometry (HPLC‐MS). We evaluated metabolite changes in whole foxtail millet seedlings treated with 200 mM NaCl in the presence or absence of JPT10 for 6 and 24 h. A total of 438 metabolites with significant changes were identified (Table S1). To find convincing differences between the metabolites, we used principal component analysis (PCA) based on metabolite levels. The five sample groups were clustered into five distinct areas in the PCA score plot, indicating that each sample group displayed unique metabolic profiles (Figure S3). PCA revealed that all samples were successfully separated, indicating significant metabolic diversity between different groups. There are many large groups of metabolites, including 45 flavonoids, 67 phenolic acids, 63 lipids, 68 alkaloids, 43 organic acids, 45 amino acids and derivatives, 13 lignans and coumarins, 49 nucleotides and derivatives, three quinones, three tannins, eight terpenoids, and 31 others (Figure S4a). They were classified as primary metabolites, secondary metabolites, and plant hormones. Primary metabolites included sugar, lipids, amino acids and derivatives, nucleotides and derivatives, and most organic acids. Secondary metabolites included flavonoids, phenolic acids, alkaloids, lignans, and coumarins. These findings imply that JPT10 controls many categories of primary and secondary metabolites. There are 84 altered metabolites at 6 h and 85 metabolites changed at 24 h, while only 20 metabolites were found to be simultaneously altered at two time points (Figure S4b, Table S1). Among the 438 metabolites with significant changes, we found that the contents of 10 flavonoids decreased while four flavonoids increased when treated with the combination of JPT10 for 6 h compared with salt stress, as well as nine phenolic acids decreased while five5 increased (Figure S5a). The obvious changes of flavonoids and phenolic acids were also observed when treated with the combination of JPT10 for 24 h compared to salt stress (Figure S5b). These results indicate the importance of flavonoids and phenolic acids in promoting salt tolerance by JPT10 in foxtail millet seedlings.

### 2.3 Exogenous AP and CGA treatments enhance the salt tolerance of millet seedlings

**FIGURE 3:**
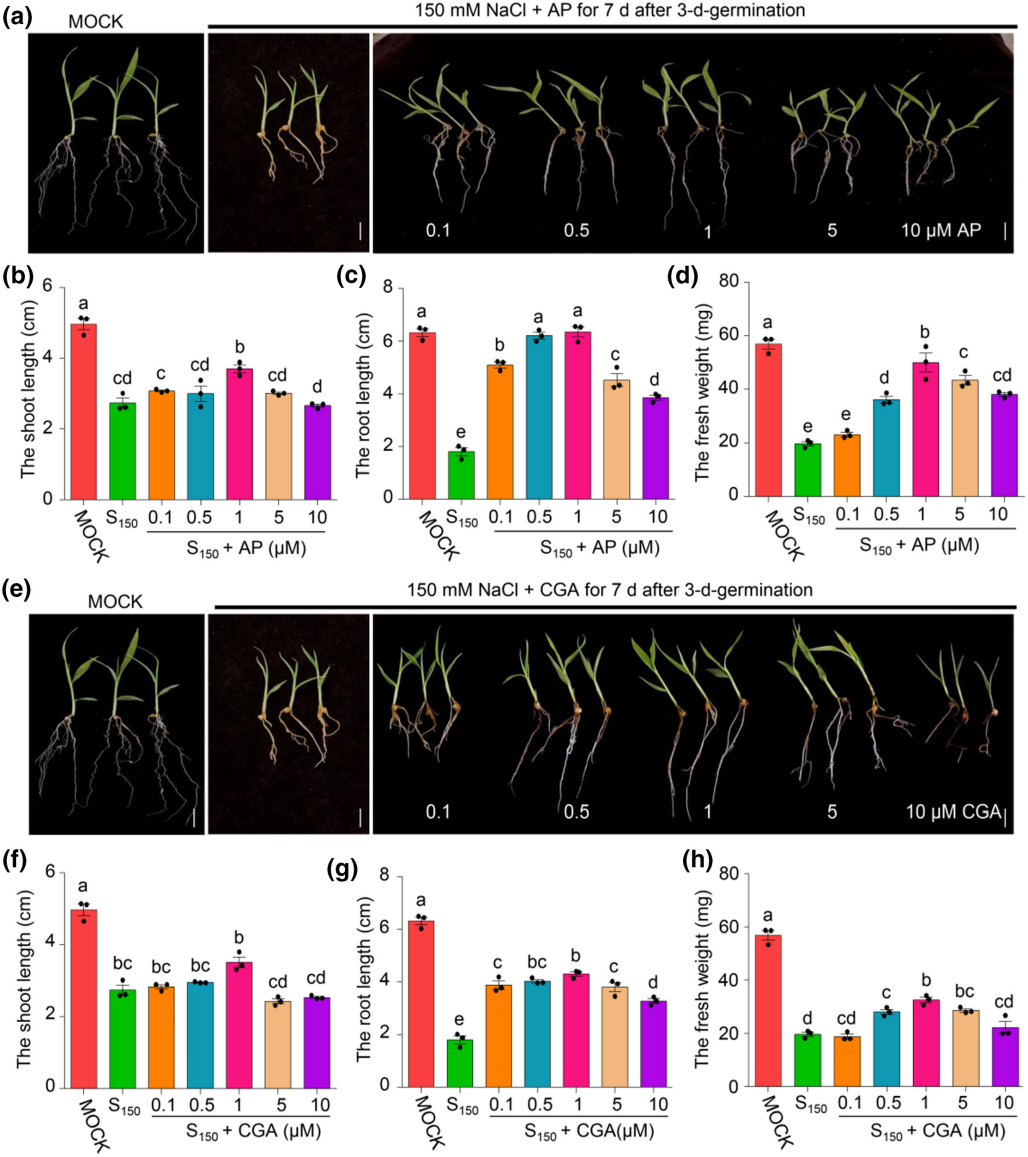
Exogenous apigenin and chlorogenic acid treatments enhance salt tolerance of foxtail millet seedlings. (a) Phenotypes of seven‐day‐old foxtail millet seedlings treated with 150 mM NaCl (S150) with or without the indicated concentrations of apigenin (AP). Seedlings treated with sterile water were used as control (MOCK). Scale bar = 1 cm. (b–dD) The shoot length, root length, and fresh weight of seedlings treated as described in (a). Error bars indicate SEM (*n* = 3). (*p* ≤ .05; one‐way ANOVA). (e) Phenotypes of 7‐day‐old foxtail millet seedlings treated with 150 mM NaCl with or without the indicated concentrations of chlorogenic acid (CGA). Seedlings treated with sterile water were used as control (MOCK). Scale bar = 1 cm. (f–h) The shoot length, root length, and fresh weight of seedlings treated as described in (e). Error bars indicate SEM (*n* = 3). (*p* ≤ .05; one‐way ANOVA).

We examined the impact of AP and CGA, representative antioxidants belonging to flavonoids and phenolic acids, respectively, on the salt tolerance of foxtail millet seedlings. Three‐day‐old foxtail millet seedlings were treated with the indicated amounts of AP or CGA for 7 days. Under salt stress, foxtail millet seedlings grew better when exposed to certain amounts of AP than when exposed to salt stress alone (Figure 3a). AP‐treated seedlings showed noticeably longer shoots and roots than those of AP‐treated seedlings under salt stress (Figure 3b,c). The fresh weight of foxtail millet seedlings in response to the combination of salt stress and AP was higher than that of seedlings treated with salt stress alone (Figure 3d). CGA showed similar effects in promoting salt tolerance of foxtail millet seedlings as AP (Figure 3e–h). The results showed that 1 μM AP or CGA was the best among the indicated concentrations for promoting the salt tolerance of foxtail millet seedlings. Furthermore, under salt stress, the germination of foxtail millet seeds and early seedling growth were both considerably enhanced by 1 μM AP and CGA (Figure S6). These results demonstrate that certain exogenous concentrations of AP and CGA can improve salt tolerance of foxtail millet.

Then, we detected that the effect of JPT10 on exogenous AP and CGA increased seed germination of foxtail millet. The results showed that salt stress inhibited seed germination was partially recovered by JPT10 and exogenous AP and CGA (Figure S7). The combination of JPT10 and exogenous AP improved salt tolerance of foxtail millet seedlings better than exogenous AP alone, but not better than JPT10 alone at all time points, and the combination of JPT10 and exogenous CGA improved salt tolerance of foxtail millet seedlings better than JPT10 alone before 72 h, whereas improved salt tolerance of foxtail millet seedlings as the same as JPT10 alone at 72 h (Figure S7). These data indicate that JPT10 improves salt tolerance of foxtail millet partially through inducing the accumulation of AP and CGA in foxtail millet especially under long‐term salt stress.

### 2.4 Multiple hormones are involved in the regulation of JPT10‐promoted salt tolerance of foxtail millet seedlings

**FIGURE 4:**
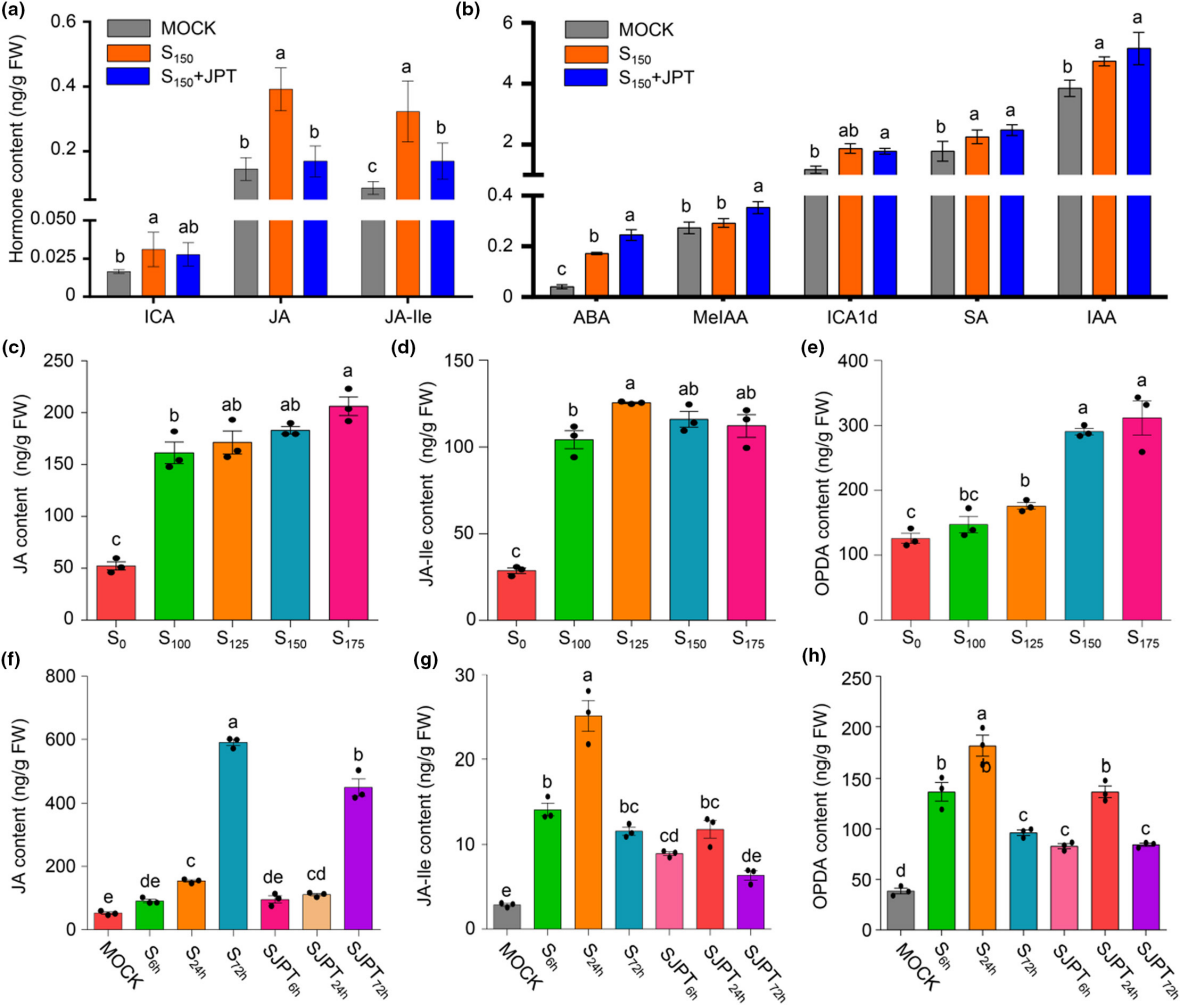
JPT10 affects the contents of multiple hormones in foxtail millet seedlings under salt stress. (a, b) Levels of multiple hormones detected in 7‐day‐old foxtail millet seedlings treated with 150 mM NaCl (S150) with or without JPT10 for 6 h. Seedlings treated with sterile water were used as control (MOCK). Error bars indicate SEM (*n* = 3). (*p* ≤ .05; one‐way ANOVA). (c–e) Contents of JA, JA‐Ile and oxo‐phytodienoic acid (OPDA) in 7‐day‐old foxtail millet seedling treated with 100, 150, 175 mM NaCl (S_100_, S_150_, S_175_) or not (S_0_). Error bars indicate SEM (*n* = 3). (*p* ≤ .05; one‐way ANOVA). (f–hH) Contents of JA, JA‐Ile and OPDA in 7‐day‐old foxtail millet seedling treated with 150 mM NaCl in the presence or absence of JPT10. S_6h_, S_24h_ or S_72h_ indicated 150 mM NaCl treatment for 6, 24, and 72 h, respectively. SJPT_6h_, SJPT_24h_ or SJPT_72h_ indicated 150 mM NaCl treatment in the presence ofJPT10 for 6, 24, and 72 h, respectively. Error bars indicate SEM (*n* = 3). (*p* ≤ .05; one‐way ANOVA).

In addition, metabolome analysis demonstrated that multiple plant hormones are implicated in JPT10‐mediated salt tolerance. The levels of ABA, SA, IAA, and their derivatives rose following JPT10 inoculation under salt stress (Table S1). To learn more about these changes, a hormonal metabolome was performed (Table S2 ). When paired with salt stress, JA and JA‐Ile formed a group with higher levels under salt stress but lower levels under normal conditions and a combination of salt stress and JPT10 (Figure 4a). The second group was composed of ABA, SA, IAA, and its derivatives, including indole‐3‐carboxylic acid (ICA), methyl indole‐3‐acetate (MeIAA), and indole‐3‐carboxaldehyde (ICA1d), with levels rising under salt stress and even further under the combined treatment of salt stress and JPT10 (Figure 4b). The third group, including gibberellins (GAs), such as GA1 and GA15, and cytokinins, such as N^6^‐isopentenyladenine (IP) and dihydrozeatin (DZ), remained unaltered under all three situations (Figure S8). These findings suggest that JPT10 increases the ability of foxtail millet seedlings to tolerate salt stress by controlling a variety of hormonal reactions.

### 2.5 JPT10 inhibits the overaccumulation of OPDA, JA, and MeJA to promote salt tolerance in foxtail millet seedlings

Different from the above hormones, JA and JA‐Ile contents were lower in JPT10‐inoculated seedlings than in JPT10 non‐inoculated seedlings under salt stress, but were higher than those in the control seedlings (Figure 4c–e). These changes were more consistent with the JPT10‐mediated salt tolerance of foxtail millet seedlings. To look more closely at how JA, JA‐Ile, and their precursor OPDA changed, their levels were measured. With increasing NaCl concentrations (Figure 4c–e), the levels of JA, JA‐Ile, and OPDA were obviously increased, which was consistent with the increasing growth inhibition of foxtail millet seedlings (Figure 1a‐d. Moreover, with prolonged salt stress, their levels rose regardless of the presence or absence of JPT10, but the presence of JPT10 was associated with levels lower than with salt stress alone (Figure 4f–h). These results indicate that JPT10 stimulates foxtail millet growth under salt stress by inhibiting JA, JA‐Ile, and OPDA over accumulation.

**FIGURE 5:**
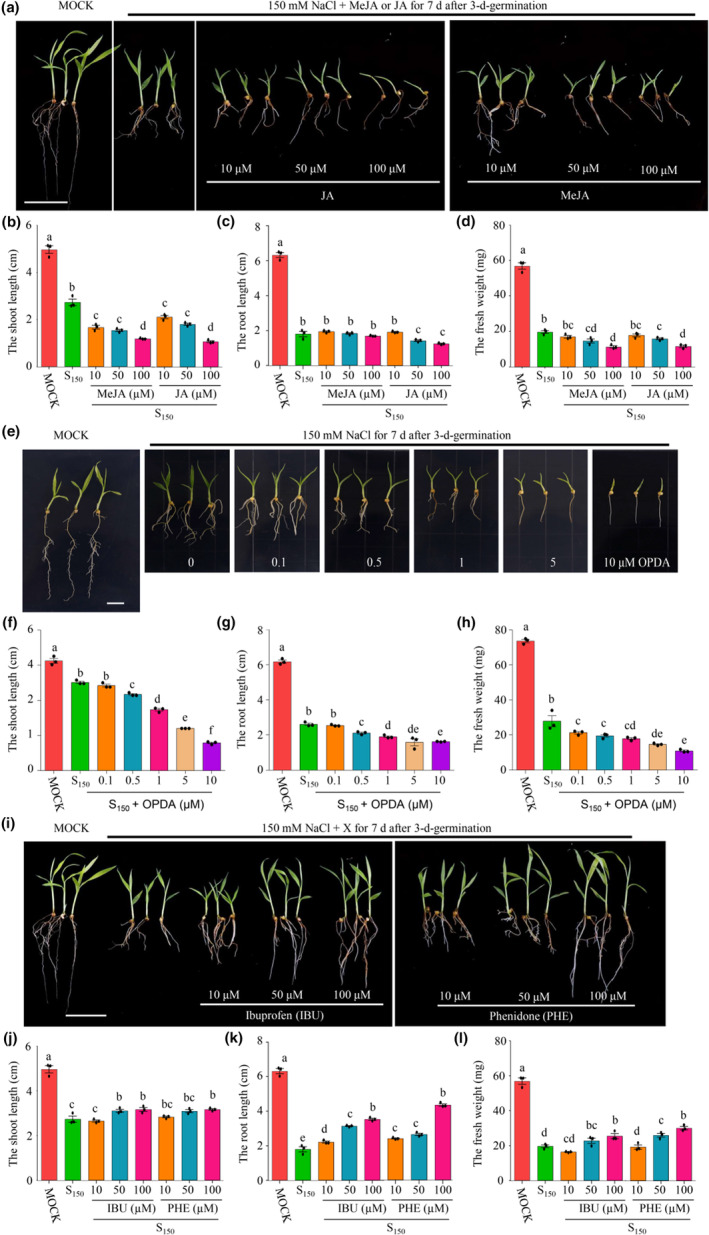
JA and OPDA intensify the salt stress to foxtail millet seedlings. (a) Phenotypes of 7‐day‐old seedlings treated with 150 mM NaCl (S150) with or without the indicated concentrations of JA or MeJA. Seedlings treated with sterile water were used as control (MOCK). Scale bar = 1 cm. (b–d) The shoot and root length, and fresh weight of seedlings treated as described in (a). Error bars indicate SEM (*n* = 3). (*p* ≤ .05; one‐way ANOVA). (E) Phenotypes of 7‐day‐old seedlings treated with 150 mM NaCl (S150) with or without the indicated concentrations of oxo‐phytodienoic acid (OPDA). Seedlings treated with sterile water were used as control (MOCK). Scale bar = 1 cm. (f–h) The shoot and root length, and fresh weight of seedlings treated as described in (e). Error bars indicate SEM (*n* = 3). (*p* ≤ .05; one‐way ANOVA). (i) Phenotypes of 7‐day‐old seedlings treated with 150 mM NaCl (S150) with or without the indicated concentrations of Ibuprofen (IBU), or phenitone (PHE). Seedlings treated with sterile water were used as control (MOCK). Scale bar = 1 cm. (j –l) The shoot and root length, and fresh weight of seedlings treated as described in (i). Error bars indicate SEM (*n* = 3). (*p* ≤ .05; one‐way ANOVA).

Therefore, we treated foxtail millet seedlings with exogenous JA, MeJA, and OPDA under salt stress. Foxtail millet seedlings underwent severe growth inhibition due to salt stress when high concentrations of JA, MeJA, or OPDA were added (Figure 5a,e). This resulted in shorter shoots and roots and lower fresh weight under the combined treatments of salt stress and high concentrations of JA, MeJA, or OPDA (Figure 5b–d, f–h). Compared to JA or MeJA, the same quantity of OPDA demonstrated a more pronounced inhibitory effect on foxtail millet seedlings. Furthermore, two exogenous inhibitors of JA and OPDA, ibuprofen (IBU) and phenidone (PHE), were applied. Foxtail millet seedlings grew better under salt stress, with longer shoots and roots and a higher fresh weight when IBU or PHE concentrations were higher (Figure 5i–l). The opposing effects of OPDA, JA, and their biosynthesis inhibitors on foxtail millet seedlings demonstrate that both OPDA and JA enhance the salt stress‐caused growth inhibition of foxtail millet seedlings.

### 2.6 JPT10 performs a conservative salt tolerance‐promoting role in crop species

**FIGURE 6:**
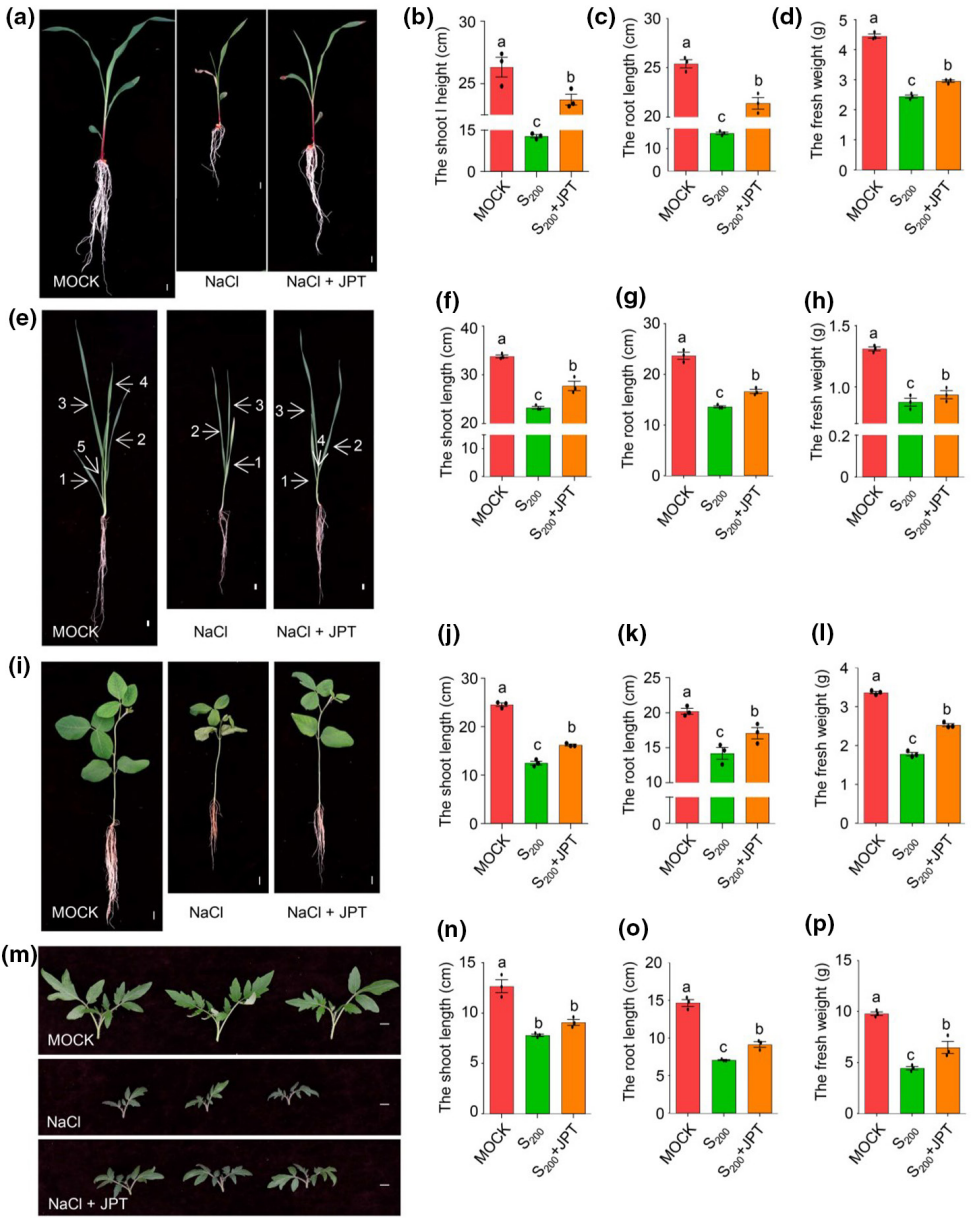
JPT10 plays a conserve salt‐tolerance promotion role in both dicots and monocots. (a–d) Growth‐promoting capacity of JPT10 in maize indicated by shoot and root length, and fresh weight. (eE–h) Growth‐promoting capacity of JPT10 in wheat indicated by shoot and root length, and fresh weight. (i–l) Growth‐promoting capacity of JPT10 in soybean indicated by shoot and root length, and fresh weight. (m–p) Growth‐promoting capacity of JPT10 in tomato indicated by shoot and root length, and fresh weight. Error bars indicate SEM (*n* = 3). (*p* ≤ .05; one‐way ANOVA).

To investigate the universality of JPT10 on crop species, JPT10 was used to inoculate soil‐grown maize, wheat, soybean, and tomato. Maize seedlings grew faster and had noticeably longer shoots and roots, and higher fresh weight under salt stress when combined with JPT10 than under salt stress alone (Figure 6a–d). Under salt stress, JPT10‐inoculated wheat, soybean, and tomato seedlings showed similar accelerated seedling growth, with noticeably longer shoot and root lengths and higher fresh weight than those that were not inoculated with JPT10 (Figure 6e–p). These findings offer compelling proof that *H. ventosae* JPT10 promotes salt tolerance in both dicots and monocots, positing this strain as a novel biological inoculum for saline agriculture.

## 3 DISCUSSION

PGPBs from naturally salty environments can be genuine candidates for more effectively reducing the impacts of salt stress on plants (Abbas et al., 2019; Bibi et al., 2019; Lucke et al., 2020; Masmoudi et al., 2019). Here, we showed that under high salt stress, *H. ventosae* JPT10 increased the salt tolerance of foxtail millet seedlings (Figure 1, Figure S2) and seed germination (Figure S7), as well as salt tolerance of seedlings from other commercially significant crop species, namely maize, wheat, soybean, and tomato (Figure 6). We also demonstrated the ideal JPT10 dosage for foxtail millet seedling growth at certain NaCl concentrations. However, JPT10 did not effectively reduce the alkaline stress that foxtail millet seedlings experience (Figure S2). These results offer guidance on how to effectively apply JPT10 to increase the salt tolerance of crops.

JPT10 effectively alleviates oxidative stress damage to foxtail millet seedlings under salt stress. We discovered that, when exposed to salt stress, foxtail millet seedlings (yugu1 cultivar) accumulated more ROS, including H_2_O_2_ and O_2_^−^, and the membrane lipid peroxidant products MDA (Figure 2a–f), indicating oxidative stress damage to foxtail millet seedlings under salt stress. The results are consistent with previous reports, which showed that Lepakshi (a salt‐sensitive foxtail millet variety), Prasad (a salt‐tolerant variety) (Sudhakar et al., 2015), and Yugu 2 (Pan et al., 2020) experienced greater H_2_O_2_ and O_2_^−^ accumulation in response to salt stress. However, JPT10 decreased H_2_O_2_ and O_2_^−^ as well as MDA overaccumulation under salt stress (Figure 2a–f), indicating less oxidative stress in JPT10‐inoculated seedlings than non‐inoculated ones under salt stress.

It has been reported that PGPBs protect plants through increasing the antioxidant ability (Ali et al., 2021; Giannelli et al., 2023; Gururani et al., 2013; Jaleel et al., 2007; Kumar et al., 2021; Kumawat et al., 2022; Singh et al., 2020). Plants have evolved two key defense mechanisms against the accumulation of excessive ROS: enzymatic breakdown by SOD, CAT, APX, and other peroxidases (POXs), and antioxidants by small molecules, such as glutathione (GSH), ascorbate acid (AsA), phenolic substances, flavonoids, and carotenoids (Pan et al., 2020). As we reviewed recently, the ability of foxtail millet to withstand salt stress is facilitated by the increased activities of SOD, CAT, and APX and transcription levels of their coding genes, as well as the accumulation of antioxidants (Wu et al., 2023). JPT10 alleviates oxidative stress damage to foxtail millet seedlings under salt stress exhibiting a similar antioxidant mechanism. This is based on the higher antioxidant enzyme activities in JPT10‐infected foxtail millet seedlings under salt stress (Figure 2g–k) and a higher content of antioxidants in JPT10‐inoculated seedlings than in non‐inoculated ones under salt stress (Figure S4, Table S1). The ability of exogenous AP and CGA in alleviating the growth inhibition of foxtail millet seedlings under salt stress was further demonstrated (Figure 3). Strong antioxidants, such as different flavonoids and phenolic substances, can mediate ROS scavenging (Hodaei et al., 2018; Kiani et al., 2021). The tomato plants *are* mutant, which have a flavonol 3‐hydroxydase (F3H) deficiency and exhibit reduced flavonol content and increased ROS formation in pollen grains (Muhlemann et al., 2018), illustrating the involvement of flavonoids in controlling ROS scavenging. Our results also showed that higher enzyme activities of SOD, POD, and APX under salt stress in the present of JPT10 than in the absent of it (Figure 2g–i). Our findings showed that JPT10 encourages antioxidant production and antioxidant enzyme activities to lessen excessive ROS accumulation under salt stress.

Moreover, JPT10 may support osmotic equilibrium in foxtail millet seedlings during salt stress. PGPBs protect plants from salt stress damage through osmotic adjustment (de Andrade et al., 2023; Giannelli et al., 2023; Kumar et al., 2021; Kumawat et al., 2022; Ramasamy & Mahawar, 2023). The bacterial regulation of the concentration of trehalose, proline, and other osmolytes in plants is one of the mechanisms behind induced plant tolerance to desiccation (Ali & Khan, 2021; Sandhya et al., 2010; Yang et al., 2010). Trehalose functions as a xeroprotectant in plant and bacterial cells through water replacement, glass formation, and stability theory to enable them to deal with severe abiotic stress, such as desiccation and/or high saline levels (Chaplin, 2006; Julca et al., 2012). *Bacillus* sp. strain wp‐6 caused salt tolerance in wheat due to 7.48%, 12.34%, and 4.12% increases in proline, soluble sugar, and soluble protein levels, respectively (Zhao et al., 2022). Our metabolomic investigation revealed that JPT10 increased the levels of trehalose, amino acids, sugars and their derivatives (Table S1). These results indicate that JPT10 increases osmoprotection to foxtail millet seedlings under salt stress.

In addition, hormone production is another important mechanism for PGPBs to protect plants against salt stress (de Andrade et al., 2023; Giannelli et al., 2023; Kumar et al., 2021; Kumawat et al., 2022; Ramasamy & Mahawar, 2023). Our findings support the notion that ABA influence the response to salt stress in foxtail millet since the elevated levels of them were caused by the combination of JPT10 and salt stress rather than salt stress alone (Figure 4b). PGPR improve plant tolerance to osmotic stress by regulating ABA biosynthesis or translocation (Belimov et al., 2014; Dodd et al., 2010). The positive roles of ABA in plant salt tolerance have been well‐understood. Indigenous ABA levels quickly rise in response to salt and osmotic stress, activating ABA signaling to increase ROS and antioxidant production and antioxidant enzyme activities to enhance stress tolerance (Jan et al., 2019). Salt stress increased the expression of ABA receptor PYL coding genes, including *SiPYL1* to *SiPYL4*, in foxtail millet (Zhang et al., 2021). High salinity induced the expression of genes encoding ABA responsive element (ABRE) binding (AREB) proteins in foxtail millet (Li et al., 2014). JPT10 may increase salt tolerance of foxtail millet seedlings by elevating ABA and then upregulating expression of ABA receptor PYL and AREB coding genes.

SA and IAA may increase the salt tolerance of foxtail millet. We found higher SA and IAA contents in salt stressed foxtail millet seedlings than the control in the presence of JPT10 or not (Figure 4b). SA is an endogenous phenolic compound that acts as a signal molecule to regulate plant environmental responses (Yang et al., 2023). For instance, the use of SA boosts plant photosynthesis, in addition to improving the antioxidant system and osmolyte production (Ahanger et al., 2019). SA helps facilitate nitrogen fixation and AM settings, reducing the deleterious consequences of salt stress (Palma et al., 2013). NPR1 (non‐expresser of PR genes 1), an SA‐binding protein, has been studied to play a central role to saline and oxidative stress tolerance in Arabidopsis (Jayakannan et al., 2015). SA can induce many ERFs, which bind to the GCC box/DRE element to upregulate the expression of the salt tolerant genes (Nie et al., 2018; Yang & Guo, 2018; Yu et al., 2020). In the tomato plant, *NHX1* and *SOS1* transcripts were found to be significantly altered in SA‐treated plants; SA regulated the ion transporter transcription and maintained ion homeostasis (Rao et al., 2021). Various studies have shown that under salt stress conditions, plants have reduced auxin levels and decreased auxin transporter expression (Du et al., 2012; Liu et al., 2015; Park et al., 2007; Ribba et al., 2020). The auxin receptor mutant *tir1/afb2/afb3* (TRANSPORT INHIBITOR RESPONSE 1/AUXIN SIGNALING F‐BOX2, 3) is more sensitive to salt treatment in terms of the root meristem (Iglesias et al., 2010). Salt stress lowers the expression of the auxin receptor‐encoding genes *TIR1* and *AFB2* (Iglesias et al., 2014). These results indicate that salt‐inhibited root growth is associated with reduced auxin accumulation (Liu et al., 2015) and suppressed auxin receptor expression. Therefore, higher levels of SA and IAA benefit foxtail millet seedlings from salt stress.

Most importantly, our results indicate that OPDA may play a more important role than JA in the response to salt stress in foxtail millet. The biosynthesis of OPDA and JA is known to be through the octadecanoid pathway (Ali & Baek, 2020; Han, 2017; Hou et al., 2016), in which abiotic (biotic) stimuli activate phospholipase in the plastid membrane, promoting the synthesis of linolenic acid (18:3) in plants (Hou et al., 2016; Wasternack & Hause, 2013). Linolenic acid is converted to OPDA through oxygenation with lipoxygenase (LOX), allene oxide synthase (AOS), and allene oxide cyclase (AOC). JA is then synthesized from OPDA by the activity of 12‐oxo‐phytodienoic acid reductase (OPR) and three cycles of beta‐oxidation. Jasmonate amino acid synthetase 1 (JAR1) catalyzes the reversible conversion between JA and JA‐Ile, the predominant bioactive JA (Fonseca et al., 2009) and an important compound in the JA signal transduction pathway (Wasternack & Hause, 2013). Both salt stress and the combination with JPT10 stimulated higher levels of JA, JA‐Ile, and OPDA than normal conditions (Figure 4a,c–h), suggesting JA, JA‐Ile, and OPDA‐mediated salt tolerance in foxtail millet. Consistently, under salt stress, JA and JA‐Ile levels were higher, and JA signaling was triggered in Arabidopsis and wheat after salt stress (Valenzuela et al., 2016; Zhao et al., 2014), which ultimately resulted in the inhibition of primary root growth. However, overaccumulation of JA related derivatives and OPDA seem to be harmful to plants. JPT10 inoculation caused lower levels of JA, JA‐Ile and OPDA than non‐inoculation under salt stress (Figure 4a,f–h). As a result, JPT10 inoculated foxtail millet seedlings grew better under salt stress than non‐inoculated seedlings (Figure 1). The application of OPDA, JA and MeJA led to aggravated growth inhibition caused by salt stress (Figure 5). Consistently, MeJA aggravated growth inhibition of Arabidopsis seedlings under salt stress (Chen et al., 2017). Interestingly, the application of OPDA led to more aggravated growth inhibition caused by salt stress than the same concentrations of JA or MeJA in foxtail millet (Figure 5), indicating that foxtail millet is more sensitive to OPDA than JA under salt stress. This phenomenon was reported previously. In which, tendril coiling of *Bryonia dioica* was more responsive to OPDA than to JA (Blechert et al., 1999; Stelmach et al., 1998).The Arabidopsis *opr3‐1* (*dde1*) (Chehab et al., 2011; Howe, 2001) and maize *opr7opr8* mutants accumulate OPDA, which, in turn, reduces the salt response in shoots and an exaggerated response in roots (Ahmad et al., 2019). OPDA has been shown to induce numerous genes in a COI1‐independent manner that is not induced by JA, in addition to upregulating COI1‐dependent genes that are likewise regulated by JA (Katsir et al., 2008; Ribot et al., 2008; Taki et al., 2005). Moreover, OPDA levels were more closely related to salt stress in plants (Figures 4e,h and 5). Thus, OPDA seems more crucial than JA in the response to salt stress in plants.

**FIGURE 7:**
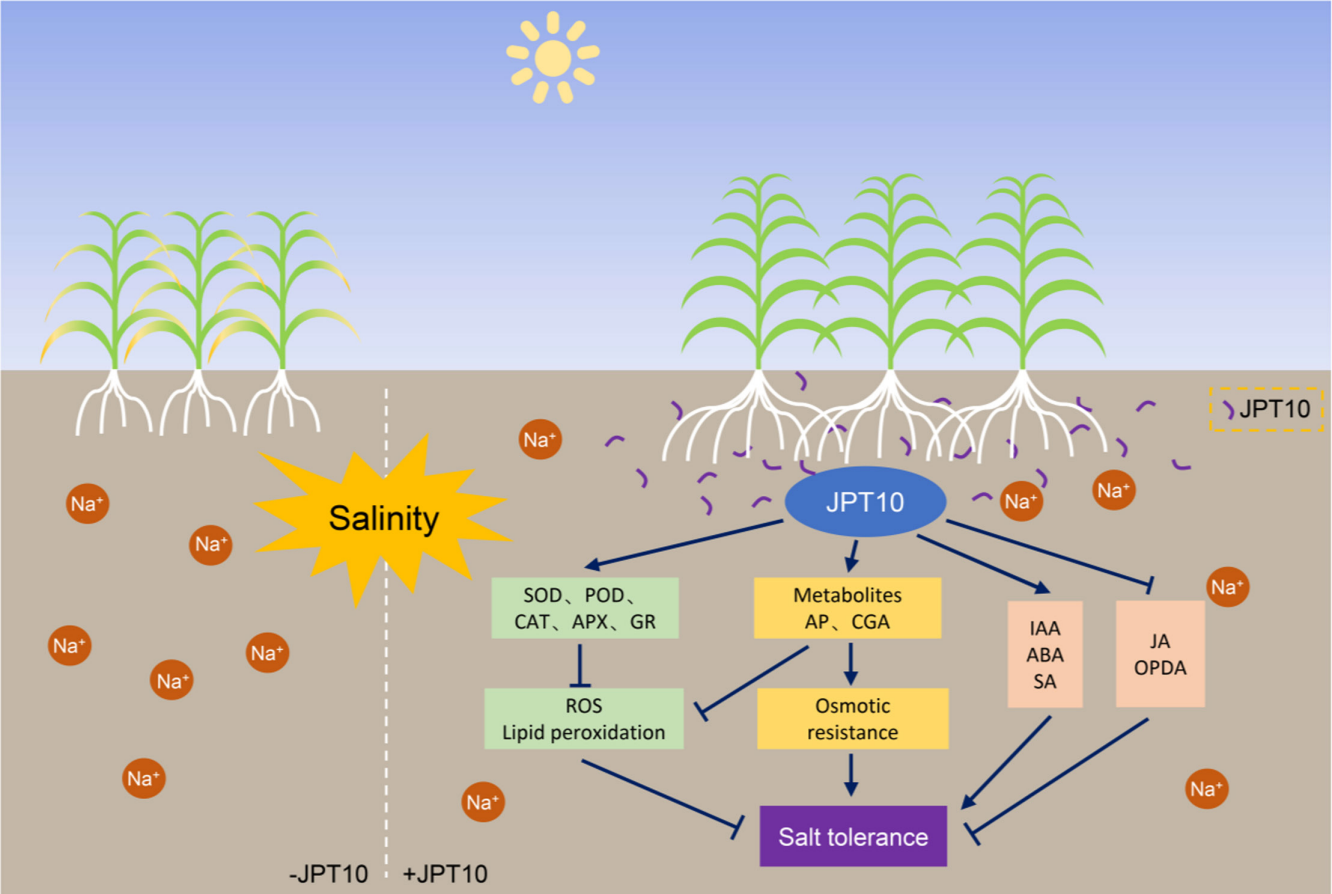
*Halomonas ventosae* JPT10‐mediated salt tolerance promotion in foxtail millet. The application of JPT10 decreased reactive oxygen species (ROS) levels by affecting antioxidant enzyme activities and increasing the contents of antioxidants and osmoprotectants, such as flavonoids, phenolic acids, glutathione (GSH), and sugars to increase the salt tolerance of foxtail millet seedlings. JPT10 treatment also enhances the levels of abscisic acid (ABA), indole‐3‐acetic acid (IAA), and salicylic acid (SA) hormones whereas decreases the levels of oxo‐phytodienoic acid (OPDA) and jasmonate (JA) to increase the salt tolerance of foxtail millet seedlings.

Together, our findings provide a physiological basis underlying the *H. ventosae* JPT10‐mediated salt tolerance promotion in crop species, including foxtail millet, maize, wheat, soybean, and tomato (Figure 7). The application of JPT10 decreased ROS levels by increasing the accumulation of antioxidants and osmoprotectants, such as flavonoids, phenolic acids, GSH, and sugars to increase the salt tolerance of foxtail millet seedlings. Moreover, JPT10 treatment enhanced the levels of ABA, IAA, and SA hormones whereas decreased the levels of OPDA, JA, and JA‐Ile to increase the salt tolerance of foxtail millet seedlings. JPT10 might alleviate OPDA and JA production by reducing lipid oxidation. However, the exact molecular mechanism underlying JPT10‐promoted salt tolerance in crops and its interaction with ROS, OPDA, hormones, and metabolites requires further investigation.

## 4 MATERIALS AND METHODS

### 4.1 Plant materials and growth conditions

Foxtail millet (*S. italica*) ‘Yugu1’ was used in this investigation. The plants were grown in a greenhouse at 25°C and 60%–70% relative humidity, with a 16 h/8 h (day/night) photoperiod. In addition, *Glycine max, Solanum lycopersicum, Triticum aestivum L*., and *Zea mays* were cultured under the same conditions.

### 4.2 Pot experiment

Endophytic *H. ventosae* JPT10 was isolated from *Suaeda salsa* growing in Kenli County, Shandong Province (37°12′26″ N, 118°48′59″ E, northern China). Luria Bertani (LB) medium with 850 mM NaCl was used for isolation. The strain has been stored in China Center for Type Culture Collection (CCTCC), the accession number is CCTCC M 2018548. JPT10 was cultured in 15 mL LB medium containing 2 M NaCl at 28°C for 24–48 h. The cultured cells were harvested and re‐suspended with water containing the indicated NaCl concentrations to 1 × 10^10^ CFU/mL.

Uniformly sized seeds of foxtail millet, soybean, tomato, wheat, and maize were selected for planting and then thinned at the two‐leaf stage. The evenly developed seedlings were placed in each designated pot (10 × 10 cm), which contained a 1:3 volume mixture of matrix and vermiculite, and every big pot contained 12 designated pots. A 2 L volume of 200 mM NaCl concentrations was irrigated in the NaCl groups, and the MOCK groups were irrigated with an equal amount of sterile water. For the +JPT10 groups, a volume of 200 mL solution containing 10^10^ CFU/mL strain JPT10 was applied to the different plants. All plants were treated every 3 days and treated three times. When the distinct phenotype manifested, pictures were taken, and data were gathered. Three biological replicates of each experiment were performed.

### 4.3 Measurement of physiological changes

The collected seedlings were washed in distilled water to remove the soil and then dried off. The fresh weight was calculated. Acetone (80%) was used to extract chlorophyll in the dark for 24 h at 25°C. The absorbance at wavelengths of 663 nm and 645 nm was assessed using a photometer (840‐210800, Thermo Fisher Scientific). The chlorophyll amount per gram of fresh weight was calculated according to a previous study (Arnon, 1949; Xu et al., 2019). To detect H_2_O_2_ and O_2_^−^, seedlings were stained using DAB (CD4181, Coolaber, Beijing, China) or NBT (N8140, Solarbio) according to previously described methods (Seligman et al., 1968). The H_2_O_2_ (BC3590, Solarbio), O_2_^−^ (BC1290, Solarbio), SOD (BC0170, Solarbio), and POD (BC0095, Solarbio) contents were detected using assay kits provided by Beijing Solabio Life Sciences & Technology Co., Ltd. The kits for APX (G0203F), CAT (G0303F), GR (G0209F), MDA (G0109F), and proine (G0111F) were bought from Suzhou Grace Biotechnology Co., Ltd.

### 4.4 Metabolome profiling

Metabolome profiling was performed utilizing a broadly targeted metabolome approach by Wuhan Metware Biotechnology Co., Ltd. As previously mentioned, sample preparation and metabolomic analysis were carried out (Chen et al., 2013). The foxtail tissues were freeze‐dried by vacuum freeze‐dryer (Scientz‐100F). The freeze‐dried sample was crushed using a mixer mill (MM 400, Retsch) with a zirconia bead for 1.5 min at 30 Hz. Dissolve 50 mg of lyophilized powder with 1.2 mL 70% methanol solution, vortex 30 s every 30 min for 6 times in total. Following centrifugation at 12000 rpm for 3 min, the extracts were filtrated (SCAA‐104, 0.22 μm pore size; ANPEL, http://www.anpel.com.cn/) before UPLC‐MS/MS analysis. Utilizing a planned protocol, the metabolites were quantified (Zhu et al., 2018). Three replicates of each sample were performed. Different metabolite values were described using a heatmap analysis with Z‐score.

### 4.5 Phytohormone determination

Fresh plant materials were harvested, weighted, immediately frozen in liquid nitrogen, and stored at −80°C until needed. Plant materials (50 mg fresh weight) were frozen in liquid nitrogen, ground into powder, and extracted with methanol/water/formic acid (15:4:1, V/V/V). The combined extracts were evaporated to dryness under nitrogen gas stream, reconstituted in 80% methanol (V/V), and filtrated (PTFE, 0.22 μm; Anpel) before LC–MS/MS analysis. JA (MeJA, JA, H2JA, and JA‐Ile), CK (IP, tZ, cZ, and DZ), auxin (IAA, ME‐IAA, and ICA), SA, and ABA contents were detected by MetWare (http://www.metware.cn/) based on the AB Sciex QTRAP 6500 LC‐MS/MS platform. Three replicates of each assay were performed.

The JA, JA‐Ile, and OPDA contents of foxtail millet seedlings were measured using the technique outlined previously (Xu et al., 2016). As for sample preparation and extraction, 100 mg fresh samples were promptly frozen in liquid nitrogen and processed. After this, samples were extracted for 3 h at 4°C using 1.8 mL of acetone and 50 mM citric acid (70:30, v/v) after being spiked with 20 μL of the stock internal standard mixture (1 μg/mL dihydrojasmonic acid and [2H5] OPDA). The extracts were kept in the dark overnight to allow the acetone to volatilize. Diethyl ether (3 × 500 μL) was used to remove the residual aqueous phase, which was then dried and reconstituted with 50 μL of methanol. For LC/MS analysis, the samples were filtered through a 0.45 μm GH Polypro (GHP, hydrophilic polypropylene) membrane NanoSep MF centrifuge tube.

### 4.6 Exogenous application of AP, CGA, JA, MeJA, OPDA, and their biosynthesis inhibitors

Yugu1 seeds were soaked in water for 12 h and germinated for 3 days. The uniformly developed seedlings were transferred to filter paper saturated with 150 mM NaCl solution with or without the indicated amounts of AP, CGA, JA, MeJA, IBU, or PHE. After 7 or 10 days of treatment, photographs were collected. The shoot height, root length, and fresh weight of the seedlings were measured simultaneously. Three biological replicates of each experiment were performed.

## 5 STATISTICAL ANALYSIS

Statistical analyses were performed using one‐way analysis of variance (ANOVA; *p* < .05; least significant difference and Duncan test) in SPSS software (version 24). All experiments had at least three biological replicates and the data were presented as means ± SEM.

## FUNDING INFORMATION

This study was funded by the Special Project of Central Government for Local Science and Technology Development of Shandong Province (YDZX2021008), the Natural Science Foundation of China (32241039), the National Key R&D Program of China (2022YFD1201704/2022YFD1201700).

## CONFLICT OF INTEREST STATEMENT

The authors declare they have no conflict of interest.

## Supporting information


Figure S1.
Figure S2.
Figure S3.
Figure S4.
Figure S5.
Figure S6.
Figure S7.
Figure S8.

Table S1.

Table S2.

## Data Availability

All data needed to evaluate the conclusions in the paper are present in the paper and/or the Supplementary Materials.
