# Supplementary material for: Halomonas ventosae JPT10 promotes salt tolerance in foxtail millet (Setaria italica) by affecting the levels of multiple antioxidants and phytohormones
Source: Plant Environ Interact. 2023 Sep 11;4(5):275–90. doi: 10.1002/pei3.10122 (PMC10564379; doi:10.1002/pei3.10122)
Supplement: Supplementary file 1 — Figure S1. Figure S2. Figure S3. Figure S4. Figure S5. Figure S6. Figure S7. Figure S8. [file PEI3-4-275-s003.docx]

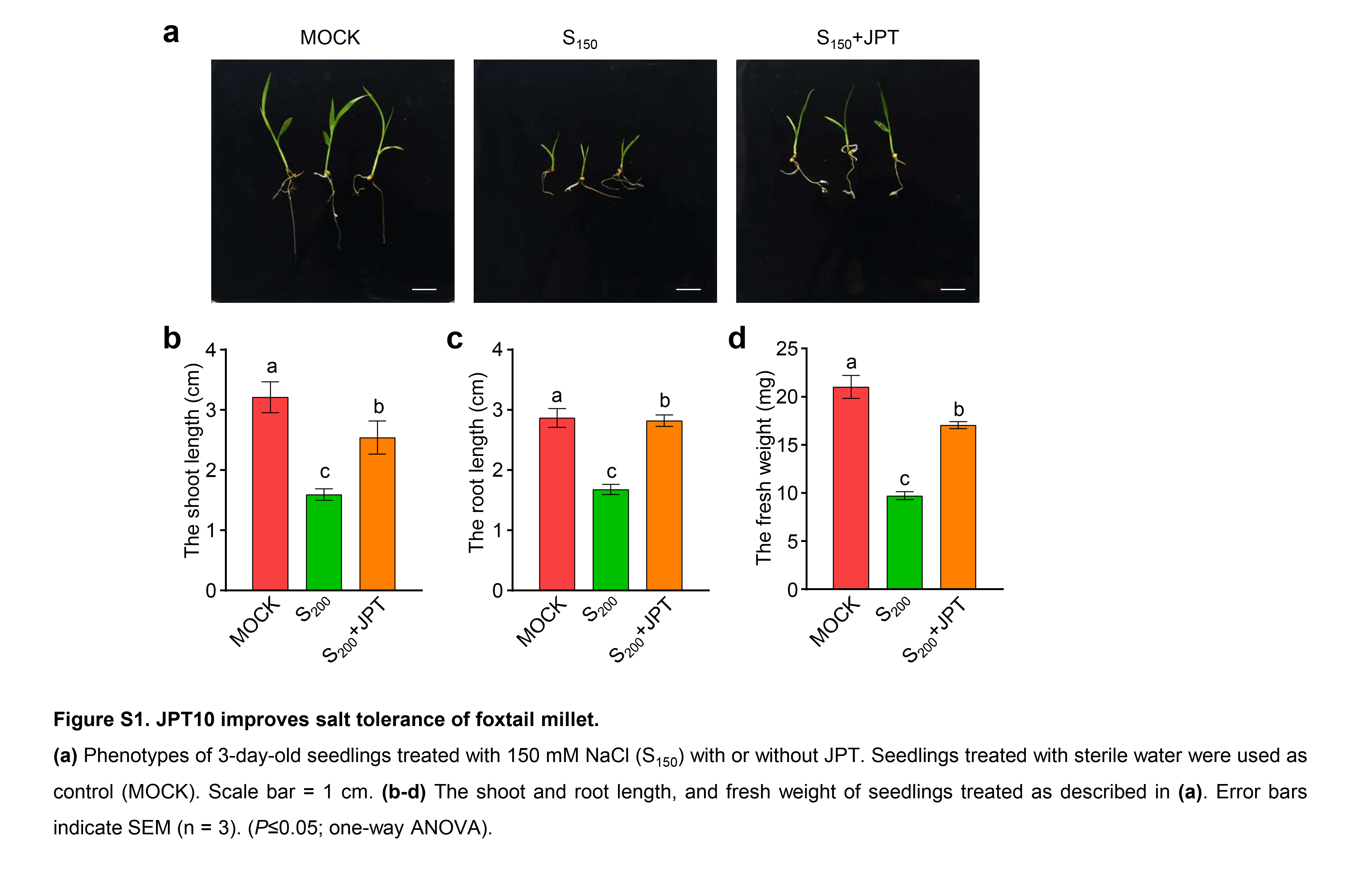
**Figure S1. JPT10 improves salt tolerance of foxtail millet.**

**(a)** Phenotypes of 3-day-old seedlings treated with 150 mM NaCl (S_150_) with or without JPT. Seedlings treated with sterile water were used as control (MOCK). Scale bars = 1 cm. **(b-d)** The shoot and root length, and fresh weight of seedlings treated as described in **(a)**. Error bars indicate SEM (n = 3). (*P*≤0.05; one-way ANOVA).


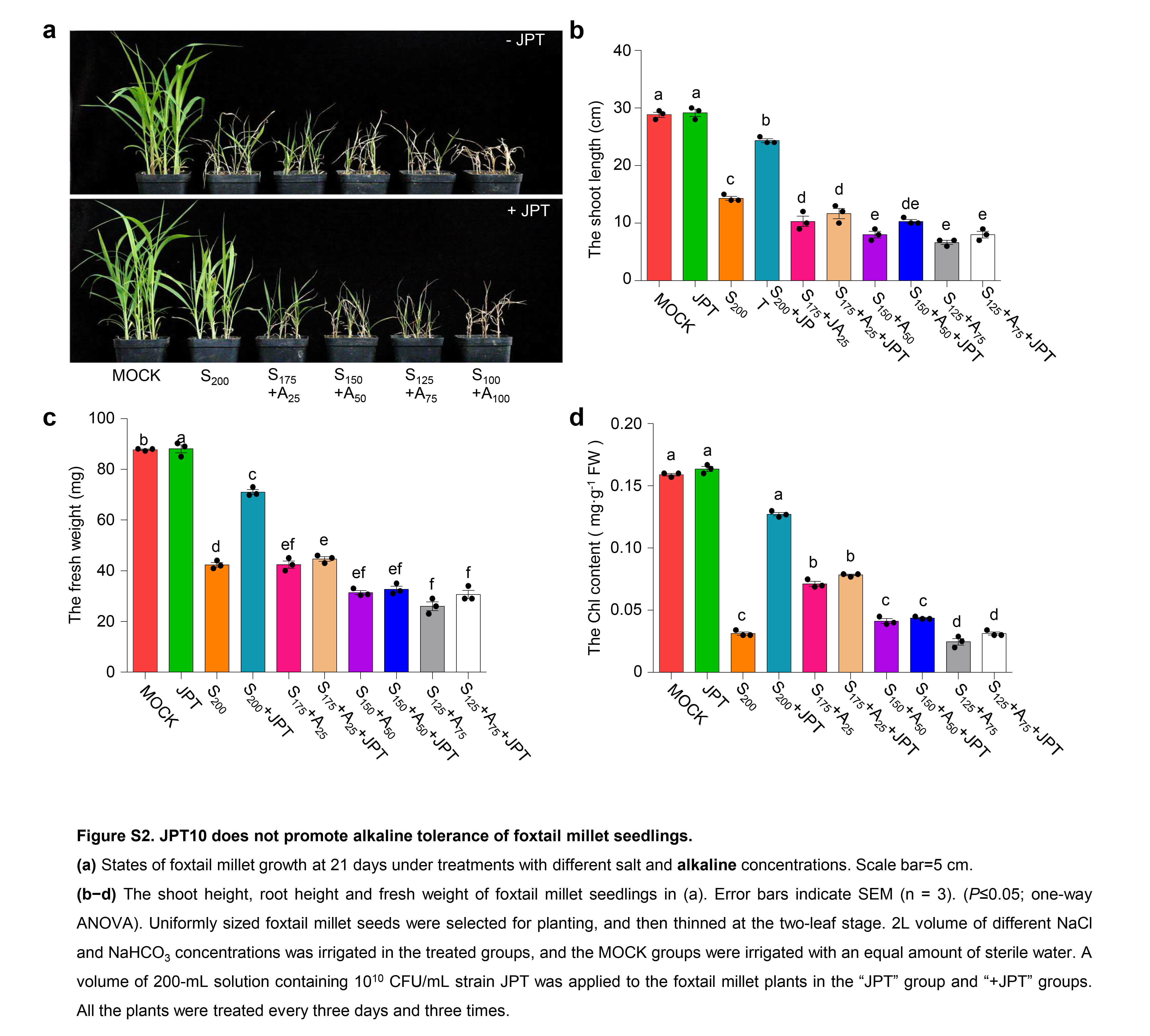
**Figure S2. JPT10 does not promote alkaline tolerance of foxtail millet seedlings.**

**(a)** States of foxtail millet growth at 21 days under treatments with different salt and alkaline concentrations. Scale bar=5 cm. **(b−d)** The shoot height, root height and fresh weight of foxtail millet seedlings in (a). Error bars indicate SEM (n = 3). (*P*≤0.05; one-way ANOVA). Uniformly sized foxtail millet seeds were selected for planting, and then thinned at the two-leaf stage. 2L volume of different NaCl and NaHCO_3_ concentrations was irrigated in the treated groups, and the MOCK groups were irrigated with an equal amount of sterile water. A volume of 200-mL solution containing 10^10^ CFU/mL strain JPT was applied to the foxtail millet plants in the “JPT” group and “+JPT” groups. All the plants were treated every three days and three times.


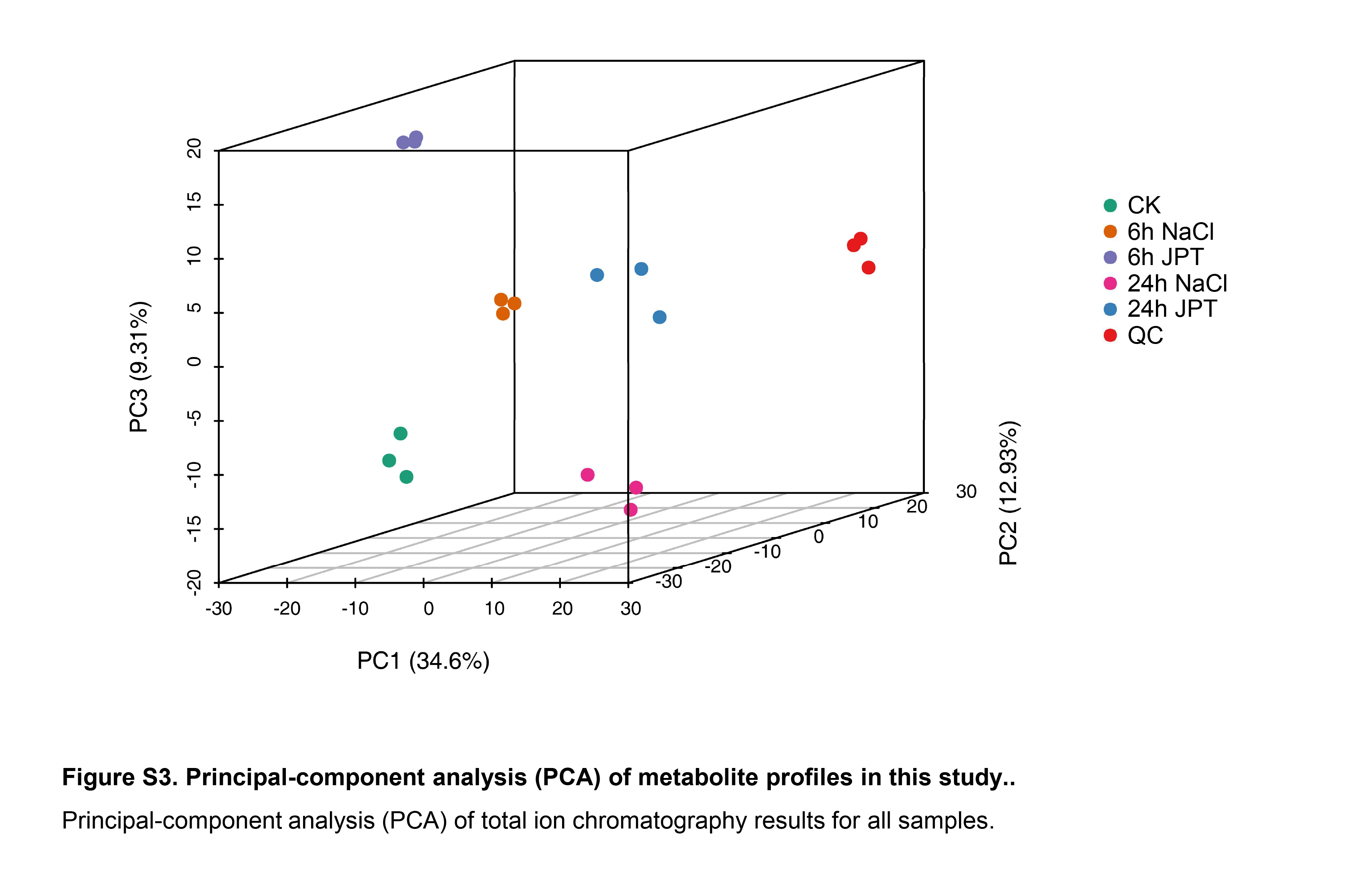
**Figure S3. Principal-component analysis (PCA) of metabolite profiles in this study.**

Principal-component analysis (PCA) of total ion chromatography results for all samples.


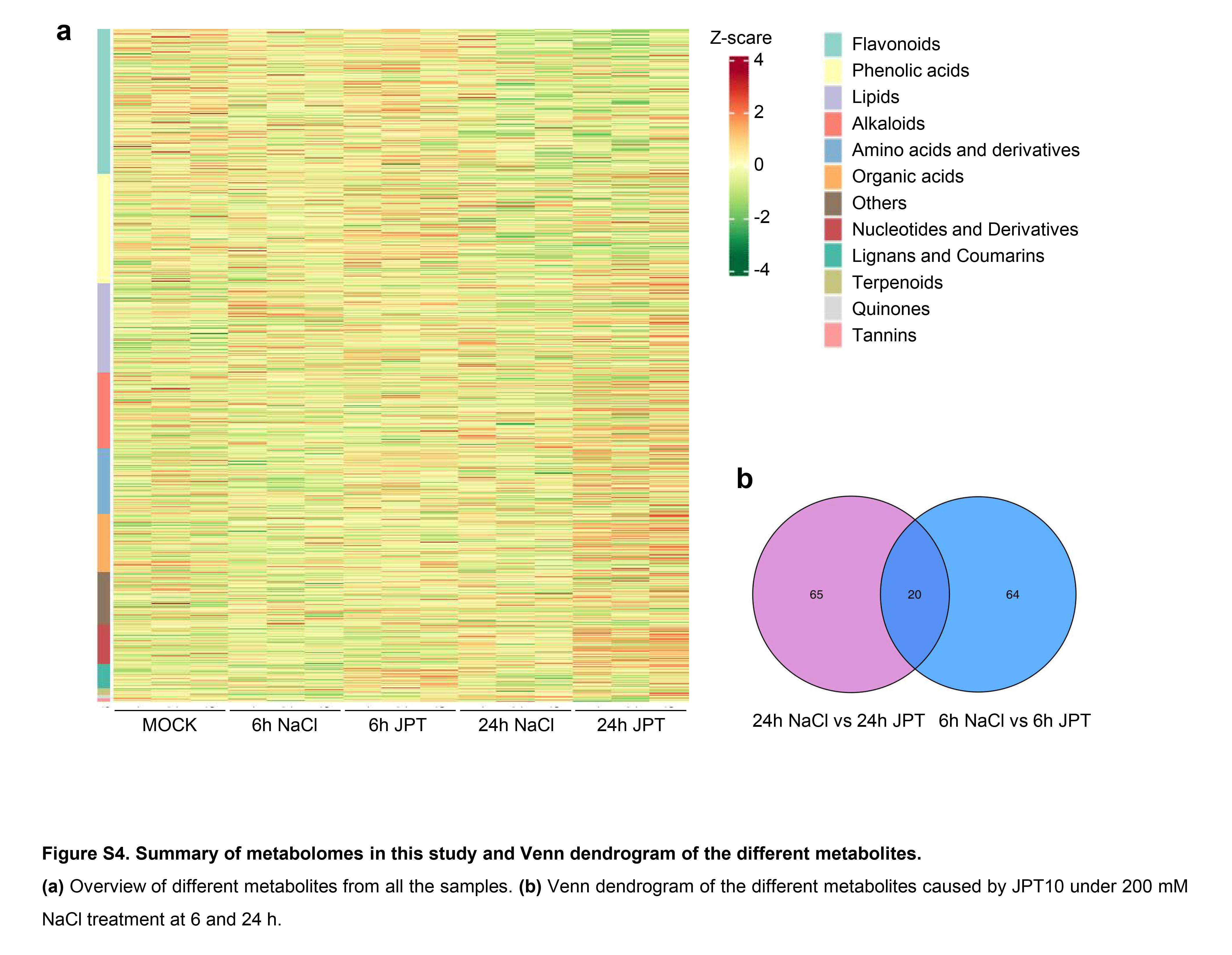
**Figure S4. Summary of metabolomes in this study and Venn dendrogram of the different metabolites.**

**(a)** Overview of different metabolites from all the samples. **(b)** Venn dendrogram of the different metabolites caused by JPT10 under 200 mM NaCl treatment at 6 and 24 h.


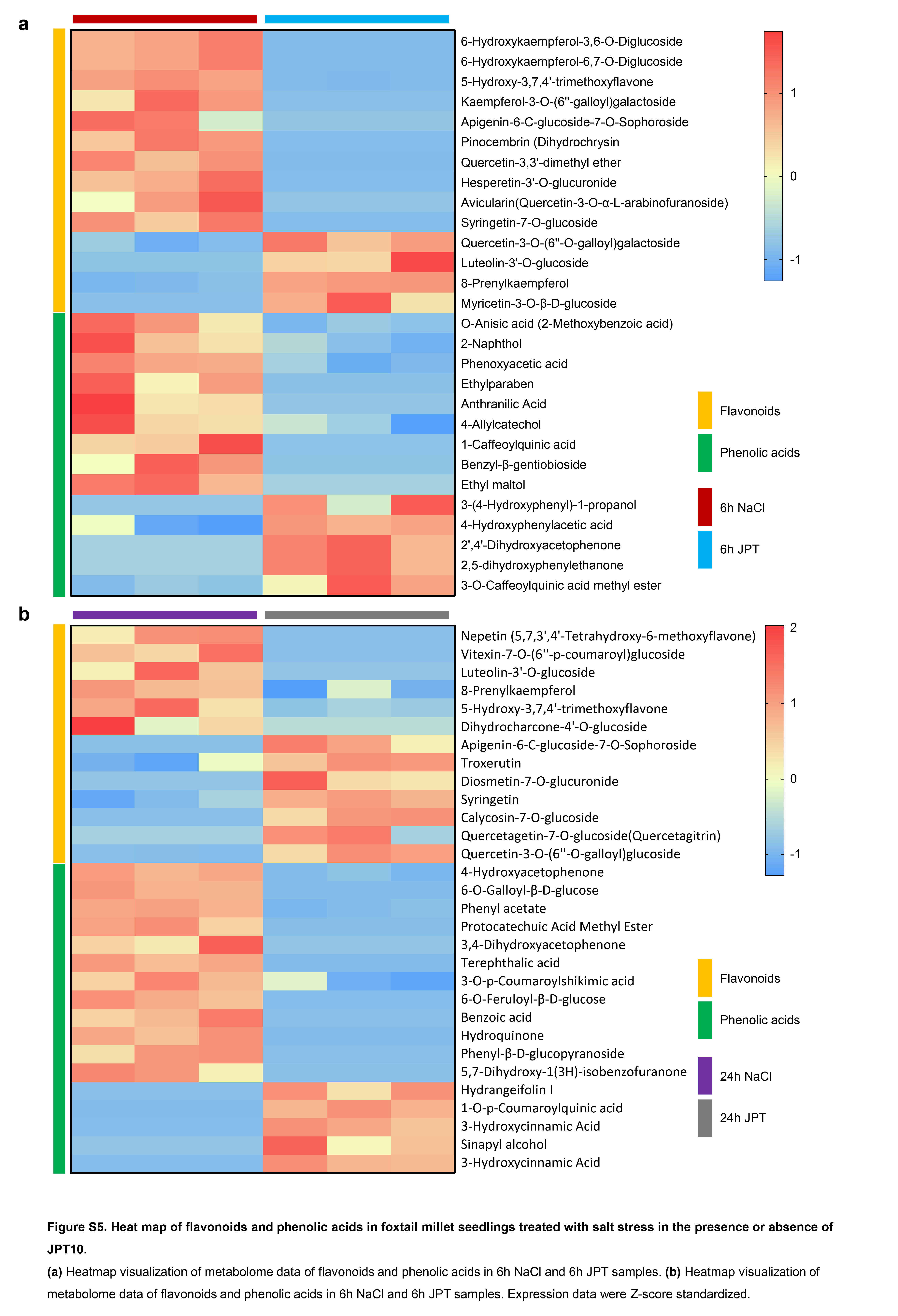
**Figure S5. Heat map of flavonoids and phenolic acids in foxtail millet seedlings treated with salt stress in the presence or absence of JPT10.**

**(a)** Heatmap visualization of metabolome data of flavonoids and phenolic acids in 6h NaCl and 6h JPT samples. **(b)** Heatmap visualization of metabolome data of flavonoids and phenolic acids in 6h NaCl and 6h JPT samples. Expression data were Z-score standardized.


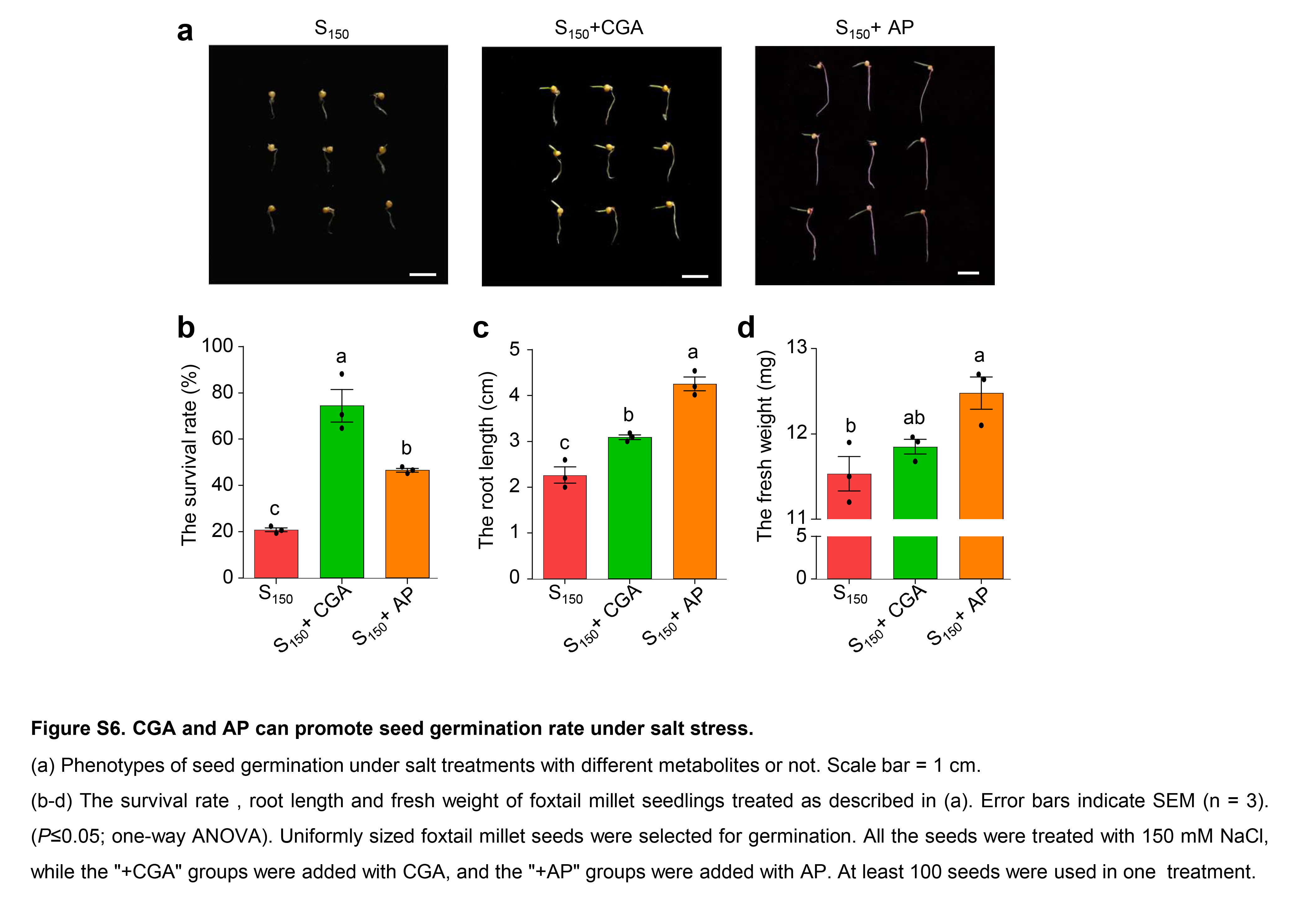
**Figure S6. CGA and AP can promote seed germination rate under salt stress.**

**(a)** Phenotypes of seed germination under salt treatments with different metabolites or not. Scale bar = 1 cm. **(b-d)** The survival rate, root length and fresh weight of foxtail millet seedlings treated as described in **(a)**. Error bars indicate SEM (n = 3). (*P*≤0.05; one-way ANOVA). Uniformly sized foxtail millet seeds were selected for germination. All the seeds were treated with 150 mM NaCl, while the "+CGA" groups were added with CGA, and the "+AP" groups were added with AP. At least 100 seeds were used in one treatment.


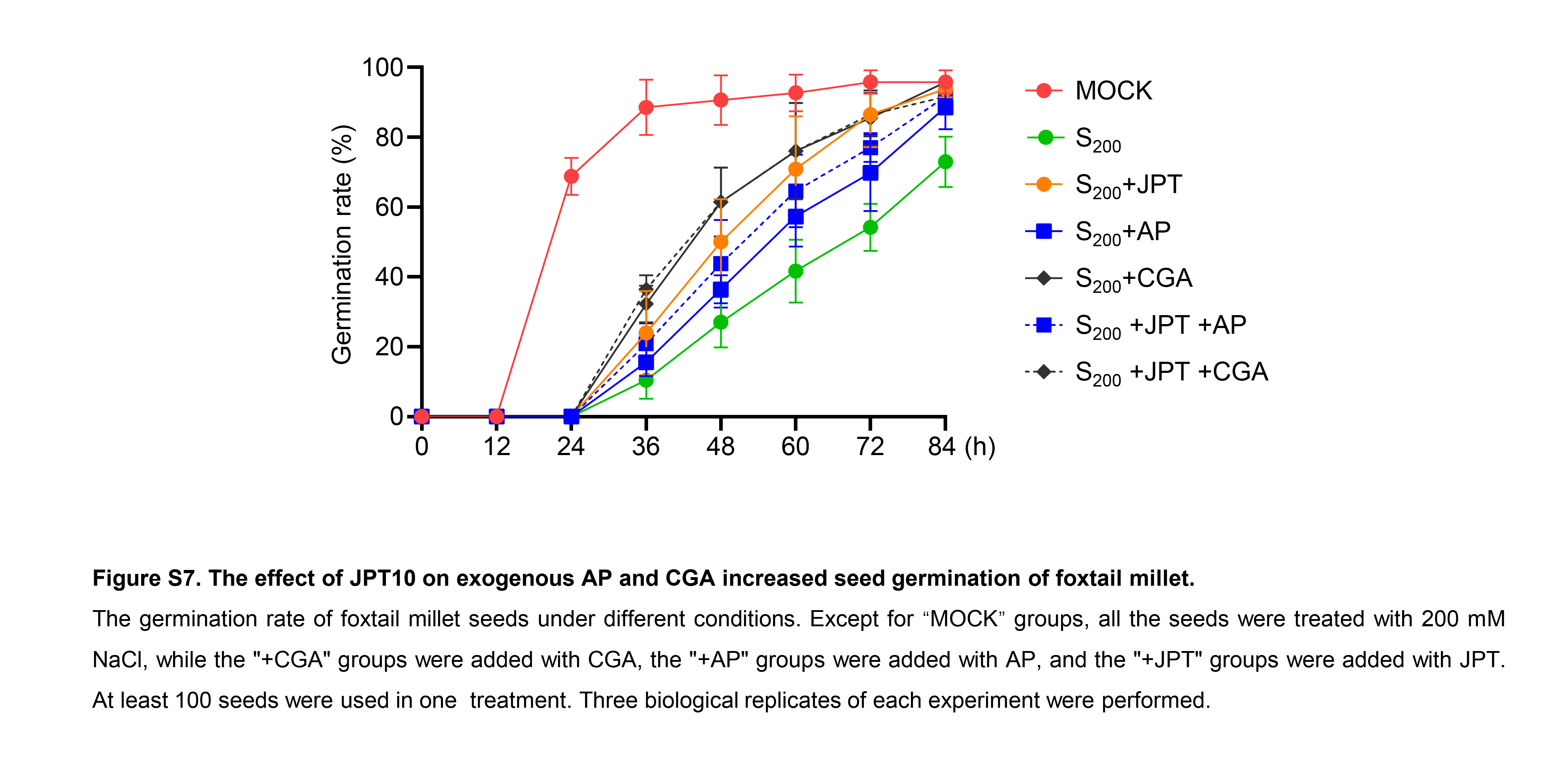
**Figure S7. The effect of JPT10 on exogenous AP and CGA increased seed germination of foxtail millet.**

The germination rate of foxtail millet seeds under different conditions. Except for “MOCK” groups, all the seeds were treated with 200 mM NaCl, while the "+CGA" groups were added with CGA, the "+AP" groups were added with AP, and the "+JPT" groups were added with JPT. At least 100 seeds were used in one treatment. Three biological replicates of each experiment were performed.


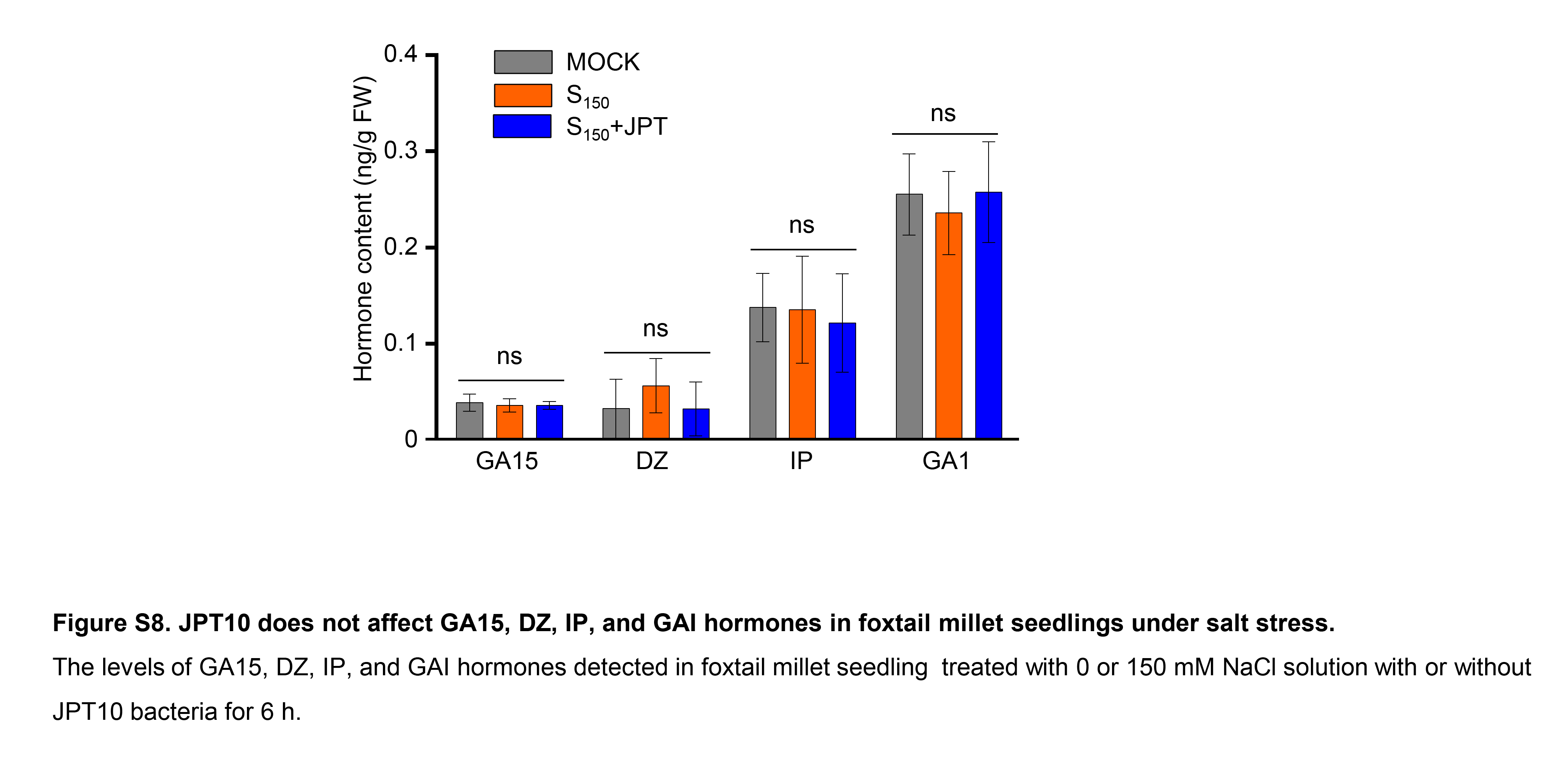
**Figure S8. JPT10 does not affect GA15, DZ, IP, and GAI hormones in foxtail millet seedlings under salt stress.**

The levels of GA15, DZ, IP, and GAI hormones detected in foxtail millet seedling treated with 0 or 150 mM NaCl solution with or without JPT10 bacteria for 6 h.
